# Odanacatib, a New Drug for the Treatment of Osteoporosis: Review of the Results in Postmenopausal Women

**DOI:** 10.4061/2010/401581

**Published:** 2010-06-14

**Authors:** José Luis Pérez-Castrillón, Florentino Pinacho, Daniel De Luis, María Lopez-Menendez, Antonio Dueñas Laita

**Affiliations:** ^1^Internal Medicine Department, Rio Hortega University Hospital, Faculty of Medicine, 47013 Valladolid, Spain; ^2^Institute of Endocrinology and Nutrition Research Support Unit, Rio Hortega University Hospital, Faculty of Medicine, 47005 Valladolid, Spain; ^3^RETICEF, 47013 Valladolid, Spain; ^4^Clinical Pharmacology Service, Rio Hortega University Hospital, Valladolid, Spain; ^5^Department of Ginecology, Hospital Rio Carrion, 34005 Palencia, Spain

## Abstract

Osteoclasts are specialized cells that initiate the process of bone resorption, which has two phases, dissolution of the mineral component and degradation of the organic matrix, in which cathepsin K plays a key role. Cathepsin K inhibitors, which block the activity of cathepsin on bone resorption lacunae, may be a new therapeutic option in osteoporosis. Odanacatib is a nonpeptidic biaryl inhibitor of cathepsin K. Two studies have evaluated the efficacy and safety of odanacatib, a phase I study to determine the dose and a phase II study of safety and efficacy. Due to the long half-life of odanacatib and the similar effects of different doses on bone remodeling markers, a weekly dosage was chosen for the phase II trail, with the best results being obtained with a dose of 50 mg. At 36 months, increases in bone mineral density similar to those produced by other powerful antiresorptive drugs (zoledronate and denosumab) were observed but there were differences in the behaviour of bone remodeling markers. Data on fractures from the phase III trial currently in development are required to confirm these possible advantages.

## 1. Introduction

Osteoporosis results from alterations in bone remodeling that cause an imbalance between bone formation and resorption, with a predominance of resorption resulting in a reduction in bone strength and the appearance of fractures. Bone remodeling is a physiological process whose function is the permanent renovation of the skeleton in order to ensure biomechanically correct bone function and the regulation of mineral homeostasis. It consists of an initial phase of bone resorption followed by a phase of formation, both of which are regulated by general (endocrine) and local (paracrine) factors. The main endocrine factors include calciotropic hormones (parathyroid hormone, and vitamin D) and sexual hormones, mainly estrogens and, to a lesser extent, androgens. Other hormones, including the thyroid hormones, growth hormone and leptin play a smaller role. Local factors include various cytokines and growth factors that regulate the process, with the inflammatory cytokines IL-1, IL-6, and TNF-*α* playing a key role [1]. The main regulator and final pathway of bone remodeling is the RANK/RANKL/OPG (Receptor Nuclear Activator Factor Kappa B/Receptor Nuclear Activator Factor Kappa B Ligand/Osteoprotegerin) system. During bone remodeling, bone marrow cells and osteoblasts produce RANKL, which binds with a transmembrane receptor of the osteoclast precursor, RANK, causing their differentiation and activation. Osteoprotegerin (OPG) is a glycoprotein that acts as a decoy receptor of RANKL, impeding the activation of osteoclastogenesis [2]. 

The most common form of osteoporosis is postmenopausal, which is initiated by a fall in estrogen levels that provokes an imbalance in the TH1/TH2 ratio (type 1 Helper T cells/ type 2 Helper T cells), with a predominance of TH1 [3]. This is caused by an increase in local levels of IL-7 which provokes increased concentrations of inflammatory cytokines and RANKL and a reduction in TGF-*β*, which exerts a beneficial effect on bone, producing an increase in osteoblastic activity and a reduction in apoptosis [4].

Osteoclasts are specialized cells derived from the mononuclear phagocyte system that initiate the process of bone resorption in two ways; dissolution of the mineral component and degradation of the organic matrix. Bone resorption begins when osteoclasts bond firmly to the bone surface through actin-rich podosomes, which form extensions of the cytoplasm to the interior of the matrix, creating specific regions named resorption lacunae. An acid medium is produced in the interior of the resorption lacunae that provokes the destruction of the osseous mineral component, leaving the organic matrix exposed. Subsequently, the organic matrix is dissolved by two enzyme groups, matrix metalloproteinases and cathepsin K, which plays a key role in degradation of the matrix [5].

Osteoporosis treatment is currently based on two drug groups, antiresorptive and anabolic agents. Antiresorptive agents, whose function is to inhibit bone resorption and generate increased bone mineral density (BMD), were the first to be introduced. The gold standard is treatment with bisphosphonates, which accelerate the apoptosis of osteoclasts and have shown their efficacy in reducing vertebral and nonvertebral fractures. However, the chronic use of bisphosphonates may result in both osseous and nonosseous undesirable effects, and this has led to the search for alternatives [6], including denosumab, which blocks the RANK/RANKL/OPG pathway [7]. Other therapeutic targets have been drugs that block integrins, which play a key role in the bonding of osteoclasts to bone, and cathepsin K inhibitors, which block the activity of cathepsin on bone resorption lacunae [8]. This article reviews current evidence for the highly selective and specific cathepsin K inhibitor, odanacatib, including results from a phase II trial.

## 2. Cathepsin K

Cathepsins are lysosomal proteases that belong to the papain-like cysteine protease family. Eleven different types have been described (B, C, F, H, K, L, O, S, V, X, and W) with cathepsin K being the most important with respect to bone remodeling, since it is a protease with intense collagenase activity, especially with respect to acid pH, which is essential to dissolve calcic hydroxyapatite, the main mineral component of bone. It degrades the two main types of collagen, I and II and is predominantly expressed in osteoclasts [9]. Immunoreactivity has also been found in osteoblasts and osteocytes although its role in these cells is not known. It is coded by a gene located in chromosome 1q21. Transcription is initiated by different regulating elements, but IL1 and RANKL can stimulate expression of the gene in osteoclasts in a process modulated by NF-kB [10]. It is a protein of 329 amino acids that consists of an amino-terminal region of 15 amino acids, a propeptide of 99 amino acids and a catalytic unit of 215 amino acids [11, 12].

The role of cathepsin K in bone resorption was determined using evidence from an autosomal recessive osteochondrodysplasia named pycnodysostosis, a very rare disease characterized by high BMD, acroosteolysis of the distal phalanxes, short stature, and cranial deformities with late closing of the fontanelles. It is caused by a genetic alteration that produces mutations of the cathepsin K gene causing loss of function [13]. Studies in mice submitted to nonfunctional mutations of cathepsin have given rise to different models of osteopetrosis. Pennypacker et al. [14] found increases in bone volume and in the number and thickness of trabecules in the distal region of the femur in a group of homozygotic cathepsin-K-null mice.

Cathepsin K is a key enzyme in the process of bone resorption and its inhibition is a new therapeutic target for the treatment of osteoporosis. The antiresorptive treatment of choice is bisphosphonates, which reduce the risk of nonvertebral and vertebral fractures. However, bisphosphonates may have adverse consequences. They increase the total number of osteoclasts, although these are described as hypernucleated, detached proapoptotic and possibly dysfunctional [15]. In rhesus monkeys, administration of odanacatib produced changes in osteoclast morphology, with the accumulation of elongated intracytoplasmic granules, although the number and size of osteoclast nuclei was not affected, indicating normal fusion. In addition the number of osteoclasts increased [16]. In addition bisphosphonates are associated with osteonecrosis of the mandible, especially in patients with tumors or diaphyseal fractures of the femur, although these adverse effects are exceptional [17–19]. The search for new therapeutic alternatives, such as cathepsin inhibitors, is interesting. The ideal inhibitor should have a low molecular weight, exhibit a minimal peptidic character, be able to bond to cathepsin and have a high selectivity to inhibit cathepsin K without affecting other cathepsins. Various inhibitors have been developed including relacatib, balicatib, MIV-701/710, and odanacatib, the object of this paper.

## 3. Odanacatib

Odanacatib is a powerful, reversible nonpeptidic biaryl inhibitor of cathepsin K that inactivates the proteolytic activity of cathepsin k. It is synthesized by replacing the P2-P3 amide bond of an aminoacetronintrile dipeptide 1 with a phenyl ring. This results in a powerful, selective inhibitor with the capacity to inhibit cathepsin K in osteoclasts. The potency and selectivity is due to the presence of the 4-fluoroleucine side chain at the P2 position interacting within the S2 pocket [8]. Its selectivity is responsible for the lack of accumulation of undesirable collagen in cutaneous fibroblasts [20]. A lack of selectivity has led to the retirement of other inhibitors in phase 2 development due to the appearance of morphea-like skin lesions [21].

Two studies have been carried out to evaluate the efficacy and safety of odanacatib, a phase I study to determine the dose and a phase II study to evaluate the safety and efficacy.

## 4. Phase I Study

This was a randomized, placebo controlled, double-blind study in post-menopausal women, without menstruation during the previous three years or during the previous year and confirmation of an elevated follicle stimulating hormone level in the postmenopausal range. The study included two groups, one containing 49 women aged ≤75 years and another containing 30 women aged ≤70 years. The objective was to evaluate the safety, tolerability, pharmacokinetics and pharmacodynamics of odanacatib in order to select the best dose. The results were measured according to the response of bone remodeling markers including CTx (carboxyterminal telopeptide of type I collagen), 1-CTP (pyridinoline cross-linked carboxyterminal telopeptide of type 1 collagen), TRAP5b (tartrate-resistant acid phosphatase), urinary deoxypyridinoline* (uDPD)*, BSAP (bone-specific alkaline phosphatase), osteocalcin, and NTx/Cr (N-terminal telopeptide of type I collagen normalized to creatinine). CTx and NTx are generated by the catalytic action of cathepsin on collagen but DPD is not influenced by the effect of odanacatib. 

The group of 49 women was used to evaluate the weekly dose. Doses of 5 mg, 25 mg, 50 mg, and 100 mg were used and 12 women were assigned to the placebo group. The group of 30 women was used to evaluate the daily dose. Doses of 0.5, 2.5, and 10 mg were used, with 6 women assigned to the placebo group. All doses were administered in fasting conditions.

Odanacatib had a long half-life of between 66 and 93 hours for all the regimes and doses used. The efficacy of weekly, and daily doses in modifying the markers was evaluated. The effect was dose-dependant although not dose proportional. Reductions in resorption markers were greatest for doses >50 mg weekly and doses ≥2.5 mg daily. Maximum suppression was achieved between day 3 and day 5 with the weekly dose and was maintained until the following dose. With the daily dose, equivalent suppression was also reached between day 3 and day 5 and remained stable whilst the drug was administered. These results suggest greater suppression with the daily dose but without significant differences with the weekly dose. Unlike other antiresorptive drugs, no effects on markers of formation, which remained at levels similar to placebo, were observed. This decoupling between markers of resorption and formation suggests a beneficial profile of odanacatib. No differences between odanacatib and placebo were observed in the number of adverse effects [22].

Due to the long half-life of odanacatib and the similar effects on bone remodeling markers between doses, the weekly dosage was chosen for the phase II trial.

## 5. Phase II Trial

This was a double-blind, randomized, placebo-controlled trial of 12 months duration with an anticipated extension period of 24 months. It included 399 post-menopausal women (no menstruation during the previous five years or bilateral oophorectomy) aged between 45 and 85 years, with a T-score <−2 but not less than −3.5 at any site. Patients were divided into five groups according to the dose: placebo, 3 mg/weekly, 10 mg/weekly, 25 mg/weekly and 50 mg/weekly. All patients received vitamin D3 (5600 U weekly) and calcium (500 mg/day in the form of calcium carbonate). The primary objective was changes in bone mass in the lumbar spine, and secondary objectives were changes in BMD in other sites, changes in bone remodeling, and adverse treatment effects.

Of 399 women randomized, 331 (83%) completed 12 months of treatment, and 320 participated in the extension study, which was completed by 270 patients (70%) at 24 months. No differences were found between women who completed or abandoned the study.

The results showed a dose-dependant increase in BMD in all sites. The greatest increase was obtained with the highest dose. Weekly administration of 50 mg of odanacatib increased bone mass by 5.7% in the lumbar spine, 4.1% in the total hip, 4.7% in the femoral neck, 5.2% in the trochanter and 2.9% in the distal third of the radius at 24 months.

Resorption markers (uNTX/Cr, sCTx, and uDPD) fell in a dose-dependant manner from the beginning of treatment and remained reduced during the first six months, after which they increased and the differences with placebo disappeared. Only the 50 mg dose showed statistically significant reductions in comparison with placebo. Bone formation markers showed no differences with placebo except for the 50 mg group, in which bone serum alkaline phosphatase (BSAP) and type I procollagen N-terminal propeptide (PINP) decreased initially but then gradually increased, with significant differences with placebo being observed for both at month 12 and month 24. Adverse effects were similar in both groups without significant differences. Bone biopsies were carried out in 28 patients and showed no adverse histologic effects [23]. The histologic effects of odanacatib were evaluated in a study carried out in ovariectomized monkeys in which the bone histomorphometry of the femoral neck was analyzed. In addition to an increase in BMD, different behaviour between cortical and trabecular bone was observed. In the trabecular bone, the behaviour was similar to established anticatabolic agents, inhibiting bone remodeling whereas in cortical bone increased bone formation was observed due to stimulation of periosteal apposition [24]. 

The results of the extension of the phase II study to 36 months have recently been reported. This included 189 women who were randomized to odanacatib 50 mg and placebo weekly. The study was completed by 169 women (89%). In the odanacatib group, BMD continued to increase (lumbar spine 7.5%, total hip 5.5%, femoral neck 5.5% and trochanter 7.4%). The urine NTX resorption marker was 50% lower compared with placebo, whereas there were no differences in the BSAP formation marker. At three years, formation markers were not only not reduced but in fact increased by 18% over baseline values. Table 1 shows the evolution of the markers. Patients in the placebo group lost bone mass although this remained above initial values and normal values of markers were re-established. This suggests that odanacatib continues to have an effect at three years and that the effect is rapidly reversible [25].

The phase I study showed the pharmacodynamics of odanacatib, with a prolonged half-life that permits weekly administration. No significant differences was observed between the daily and weekly dose in the suppression of bone resorption markers. There were no differences in adverse effects between placebo and odanacatib. Taking these data into account, the phase II trial used the weekly dosage, achieving the best effects with a dose of 50 mg. At 36 months, increases in BMD similar to those of most powerful antiresorptive agents (zoledronate and denosumab) [6, 26] were observed, but with differences in the behaviour of bone remodeling markers. Decoupling between markers of formation and resorption were observed in tandem with increases in the therapeutic window. There was a smaller reduction in markers of resorption in comparison with other powerful antiresorptive agents but, in turn, the reduction in levels of formation markers was much smaller.There are no data on fractures, a key element in demonstrating the efficacy of a drug against osteoporosis. To clarify this point, a study (ClinicalTrials.gov registration number: NCT00529373) is ongoing, with results expected in 2012. This is a clinical, randomized, double-blind, trial with 16,000 patients. The target population is postmenopausal osteoporotic women aged ≥65 years not previously treated for osteoporosis. Patients with metabolic bone diseases other than osteoporosis or with previous hip fracture will not be included. Odanacatib at a dose of 50 mg/weekly will be used and placebo will include calcium and vitamin D. The primary objective of the study is the reduction in osteoporotic fractures (vertebral, nonvertebral, and hip). 

In conclusion, odanacatib is a cathepsin K inhibitor whose mechanism of action differs from that of other antiresorptive agents. It does not reduce the number of osteoclasts and does not alter their function, thereby offering theoretical advantages over bisphosphonates. The results of the phase III trial currently in development are required to confirm these possible advantages.

## Figures and Tables

**Table 1:** Effect of 50 mg of odanacatib on formation markers and resorption at 12, 24, and 36 months.

	12 months	24 months	36 months
BSAP	−18%	−15%	+18%
NTx/Cr	−60.2%	−51.8%	−50%
CTx	−60%	−45%	−24%

NTx/Cr: N-terminal telopeptide of type I collagen normalized to creatinine.

BSAP: bone-specific alkaline phosphatase.

CTx: carboxyterminal telopeptide of type I collagen.
